# Enhancing the Strength of 3D-Printed Polymer Exoprosthetic Socket by Localized Non-Planar Continuous Carbon Fiber Reinforcement

**DOI:** 10.3390/polym17081097

**Published:** 2025-04-18

**Authors:** Daria Dolgikh, Evgeniy Lobov, Igor Bezukladnikov, Aleksandr Shalimov, Mikhail Tashkinov

**Affiliations:** 1 Laboratory of Mechanics of Biocompatible Materials and Devices, Perm National Research Polytechnic University, 29 Komsomolsky Ave., 614990 Perm, Russia; dolgikhdar@pstu.ru (D.D.); eslobov@pstu.ru (E.L.); shalimov96@pstu.ru (A.S.); 2 Department of Automation and Remote Control, Perm National Research Polytechnic University, 29 Komsomolsky Ave., 614990 Perm, Russia; corrector@pstu.ru

**Keywords:** exoprosthetic socket, continuous carbon fiber reinforcement, additive manufacturing, progressive failure analysis, multi-axis 3D-printing

## Abstract

This study investigates strategies to enhance the structural integrity of 3D-printed orthopedic transtibial exoskeleton sockets by integrating non-planar reinforcement with structured prepreg rods composed of continuous carbon fibers, leveraging multi-axis additive manufacturing techniques. A prototype of a cylindrical polyamide 3D-printed exoskeleton socket is examined. Numerical modeling using progressive failure analysis, incorporating material property degradation models, successfully simulated damage accumulation in the studied 3D-printed structures. Numerical simulations revealed that crack formation initiates in the socket’s distal section, aligning with physical test observations. Targeted localized reinforcement with carbon rods effectively strengthened the high-load regions of the prosthetic devices. A method to improve product strength by optimization of the internal architecture of the embedded reinforcements in the local stress concentrator zones is proposed. The results demonstrate a reduction in stress concentrations within prostheses when using carbon fiber reinforcements. Multi-axis dual extrusion non-planar additive manufacturing techniques were used to produce the developed prototypes. Surface morphology was examined, and optimal process parameters were determined to enhance printing quality. The developed approach enables precise reinforcement of custom-shaped sockets with complex geometries.

## 1. Introduction

Additive manufacturing has proven to be a highly effective technique for creating customized products with complex geometries, serving diverse industrial, scientific, and consumer applications. A relevant area for the application of such technologies is personalized orthopedics. Modern additive technologies provide significant topological freedom, allowing for the optimization of orthopedic prosthesis parameters to meet the individual needs of patients. This capability enables the creation of products with unique properties that perfectly match the anatomical features of each individual. An exoskeleton prosthesis for transtibial amputation consists of three main components: the socket, the pylon, and the foot [1]. The prosthetic socket is a cup-shaped structure that fits over the residual limb and transmits mechanical loads from the body to the prosthesis [2]. A 3D-printed socket can be designed using data from a comprehensive scan of the residual limb. It is the only personalized component that conforms to the shape of the limb, whereas other parts can be produced based on standard products.

The design of a socket has two main issues that can be addressed through additive manufacturing and controlled integration of reinforcing elements. The first problem is related to uneven loading on the prosthesis, leading to its failure. Studies [3,4,5] have shown that the distal end of the socket is the most vulnerable area for crack initiation. In the distal section of the socket, brittle failure is observed, with crack propagation occurring parallel to the direction of the printing [3]. The second key challenge involves ensuring the comfort and proper fit of the prosthetic socket. Wearers often experience discomfort in the soft tissues of the residual limb, particularly at the interface where it meets the prosthetic socket. Addressing this issue requires a detailed analysis and optimization of pressure distribution across the socket’s interior surface, which remains a critical aspect of advancing prosthetic design and improving user outcomes [1,6,7,8].

The idea of improving the mechanical characteristics of 3D-printed products through the addition of reinforcing elements was previously discussed in [9,10,11,12,13,14,15]. The inclusions can differ both in length (ranging from short to continuous) and in material composition (such as carbon fiber, fiberglass, quartz fibers, silica fibers, etc.). Research papers [16,17,18,19,20] have shown that polymer composites obtained by reinforcing thermoplastics with continuous fibers exhibited excellent strength properties. Furthermore, composites with continuous carbon reinforcement are lighter, possess high stiffness and wear resistance, and withstand fatigue loads. The results reported in [21] highlight the high anisotropy of carbon fibers, which allows for the creation of various structures with complex configurations. Carbon filaments, in the form of a rod, are surrounded by the same polymer matrix from which the product is made, significantly enhancing its adhesive ability. Additionally, the rod can be used in conjunction with a polymer matrix containing short fibers, thereby further increasing the strength characteristics of the structure. Another advantage of using carbon rods is that they can be integrated into the design of a socket in such a way that it does not compromise its aesthetic qualities.

Numerical modeling plays an important role in the initial evaluation of the structural integrity of the socket. The finite element method allows for the preliminary analysis of pressure redistribution that occurs when carbon rods are added into the socket structure, as well as optimizing its design, in terms of topology, and assessing changes in strength characteristics. Material properties at the component level, such as fiber orientation, influence stresses at the microscopic level [22,23]. Thus, conducting a preliminary mechanical analysis is essential for prediction of the failure process [24].

Although carbon fibers are lightweight, full reinforcement of the structure is impractical due to the significant costs associated with reinforcing material. A more rational approach is to reinforce the socket only in areas subject to high loads which are potentially critical for crack formation. This allows for optimization of the reinforcement scheme in accordance with operational requirements. The task of selecting optimal reinforcement parameters for the distal end of the socket, and assessing the mechanical characteristics of the structure after local reinforcement of the areas prone to crack initiation with carbon rods can be solved numerically.

This work aims to develop an approach for designing 3D-printed exoprosthetic sockets with controlled localized reinforcement, and to propose methods for their production using a multi-axis dual extrusion non-planar additive manufacturing technique. The research is conducted in four stages.

(i) Investigation into optimal reinforcement schemes by determination of the optimal spacing interval for carbon rod integration in rectangular polymer specimens through three-point bending tests.
(ii) Finite element modeling of the prosthetic socket with carbon rods positioned in critical stress zones identified during the first phase.
(iii) Investigation of fracture in reinforced sockets using progressive damage modeling techniques [25,26,27], observing damage accumulation and propagation under operational loads.
(iv) Manufacturing of a cylindrical prototype with localized carbon fiber reinforcement employing advantages of multi-axial 3D-printing.

## 2. Materials and Methods

### 2.1. Materials

The material used for the socket was polyamide PA12 (nylon)—a polymer with high strength and stiffness characteristics, which exhibits excellent abrasion and chemical resistance, as well as high adhesive properties. It is widely used in 3D printing in the biomedical field, allowing for the creation of complex geometric shapes and customized medical devices, such as prosthetic components [28]. Another advantage of PA12 is its low water absorption rate. This property ensures the stability of mechanical properties even in humid environments, allowing prosthetics to be used in adverse weather conditions without loss of functionality. Polyamide PA12 meets the requirements of EN ISO 10993-1 [29], a standard that validates its biocompatibility and safety for medical device applications. This certification ensures the material does not provoke adverse biological responses and maintains safe interaction with bodily tissues, making it ideal for producing devices designed for skin contact.

Polyamide PA12 filament was purchased from Anisoprint (Shanghai, China). Its mechanical properties were obtained from the analysis of the stress–strain curves obtained for tensile tests. Standard samples (according to ISO 527-2:2012) [30] with an infill angle of 90° and a printer nozzle diameter of 0.4 mm were examined. The infill angle corresponded to the printing process parameters of a socket. To obtain data on statistical variation, tests were conducted on 9 samples. The resulting properties are presented in Table 1.

The carbon fiber prepreg from F2 Innovations (Perm, Russia) with a volume fraction of carbon fibers of 60% was employed for reinforcement. A continuous carbon tow containing 3000 individual carbon filaments was consolidated into a rod with a diameter of 0.4 mm using polyamide as the binding material. Its mechanical properties were provided by the manufacturer, and are presented in Table 1.

### 2.2. Modeling and Numerical Simulation

The initial stage of the prosthetic socket design typically involves creating geometry based on the residual limb characteristics. In this study, a simplified cylindrical prototype of a socket was examined. Subsequently, numerical modeling was employed to identify regions of uneven load distribution which led to crack formation in the polymer material. Such analysis aims to optimize reinforcement parameters and to assess variations in mechanical properties. Finite element modeling using SIMULIA Abaqus 2023 was employed to determine the optimal carbon rod configuration and to study their interaction with the polymer matrix. Both the polymer matrix and carbon rods were modeled using isotropic materials.

Changes in the mechanical characteristics during damage accumulation in the polymer material can be described by Hooke’s law, taking into account the degradation of elastic properties as follows:

(1) $$\left\{\begin{array}{c} \sigma_{ij}=C\left(D_{ij}\right)\varepsilon_{ij}\\ \varepsilon_{ij}=S\left(D_{ij}\right)\sigma_{ij} \end{array}\right.,$$

According to the model, the values of the damage variables $D_{ij}$ range from 0 to 1, where a value of 1 corresponds to complete failure. The compliance matrix depends on the damage variables and is expressed as follows:

(2) SD=S^11111−D11S^1122S^1133000 S^22221−D22S^2233000  S^33331−D33000  sym. S^12121−D1200    S^23231−D230     S^13131−D13.

As soon as the stresses in a point meet the failure criterion, the stiffness is reduced to a certain value in accordance with a predefined degradation coefficient. To implement the failure simulation of the polymer material with degradation of the elastic properties, a user subroutine UMAT was used [25,26,27]. In this paper, all damage variables $D_{ij}$ for Polyamide 12 (PA12) were the same, and were equal to 0.9. This means that, after the failure criterion is reached, the material retains 10% of its original stiffness. This particular value reflects the material’s behavior under specific stress conditions, particularly its ability to withstand mechanical and thermal stresses while accounting for potential damage mechanisms [32]. Setting the degradation factor to a value less than unity also helps maintain the stability of the numerical solution and prevents the occurrence of singularities.

### 2.3. Multi-Axial Additive Manufacturing and Non-Planar Reinforcement

The cylindrical prototype was manufactured using the fused filament fabrication (FFF) 3D-printing technique. The additive manufacturing process was implemented using a custom-built multi-axis robotic cell developed by F2 Innovations (Perm, Russia), which integrates a robotic manipulator from Fanuc (Yamanashi, Japan). The robotic cell is equipped with two interchangeable printing heads: one designed for thermoplastic filament extrusion via FFF, and the other for depositing polymer-coated carbon rods.

The manufacturing parameters used for 3D-printing with PA12 included a layer thickness of 0.3 mm, an extrusion line width of 0.6 mm (100% of the nozzle diameter), a layer temperature of 80 °C, and a nozzle temperature of 265 °C. For continuous carbon fiber deposition, the parameters included a layer thickness of 0.2 mm and a nozzle temperature of 265 °C.

## 3. Results

### 3.1. Optimal Arrangement of Carbon Rods

**Figure 1:**
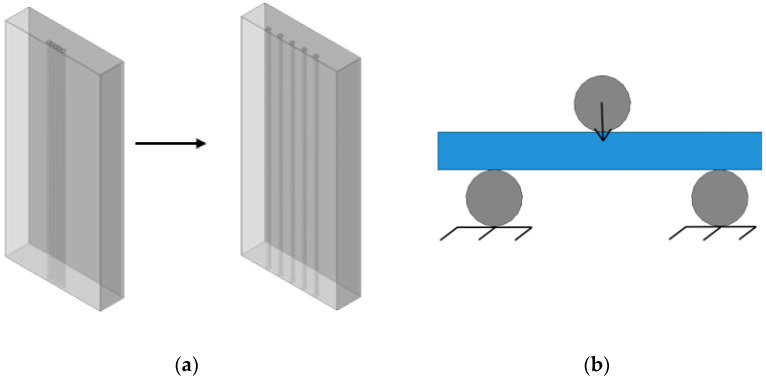
Scheme of reinforcement of polymer matrix with rods (**a**), and three-point bending test (**b**).

To investigate the optimal parameters for reinforcement, a numerical analysis of rectangular samples under three-point bending was conducted (see Figure 1a).

Samples of the polymer matrix with dimensions of 35 mm × 16.2 mm × 4 mm reinforced with five carbon rods positioned at various distances were examined. The plate thickness of 4 mm corresponded to the projected thickness of a socket. The diameter of a carbon rod was 0.6 mm. The distance between the rods was altered with an increment step of 0.1 mm, starting from the initial position where the rods were placed adjacent to each other (see Figure 1a). Ideal adhesion between the rods and the matrix was assumed.

Three-point bending boundary conditions were considered (see Figure 1b). The steel lower rollers were rigidly fixed, while the upper roller was allowed to move only along the Y-axis. To prevent displacement of the sample along the X-axis, additional contact conditions were imposed on the side surface to restrict movements in this direction and to fix rotation angles in all directions. A multi-point constraint was used to link the side surface with a rigidly fixed reference point, which helped avoid loss of stability in the plate. Ideal contact conditions were defined between the rollers and the samples. The problem was solved in terms of displacements (the load was applied through the displacement of the upper roller, initially set at 0.4 mm or 10% strain relative to the plate thickness). The optimal distance between the rods was determined based on the mechanical state of the polymer matrix.

**Figure 2:**
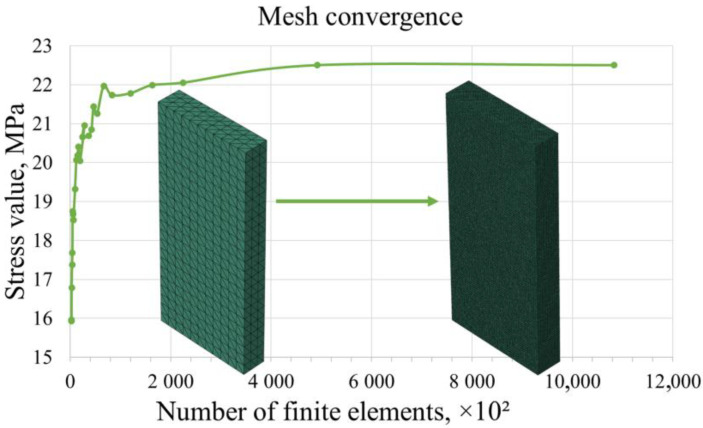
Evaluation of finite element mesh convergence in terms of mean equivalent stresses (von Mises) in a plate with respect to the total number of elements during progressive failure analysis.

To ensure the quality of the finite element mesh, the convergence of numerical results was examined by adjusting the average size of the finite elements. The size of the finite elements was progressively reduced until the maximum stress in the model stabilized at a specific value. This determined the range in which increasing the mesh density resulted in minimal changes in the stress values. A model without reinforcement was used for the convergency study (see Figure 2).

Based on the calculation results, it was found that, within the range of finite element sizes from 0.3 mm to 0.2 mm, the convergence graph approached an asymptote. Further changes to the mesh parameters were not advisable, as they may significantly increase computational time. In the models with reinforcement, the mesh was refined around the rods (with the mean size of 0.1 mm).

**Figure 3:**
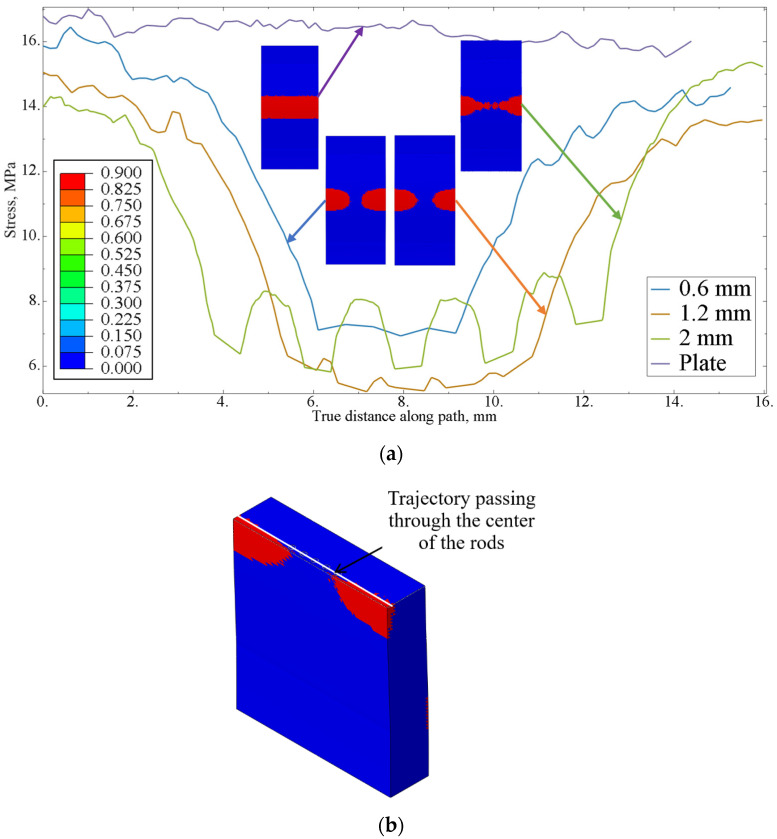
The fields of the failure criterion (**a**), and the stress distribution diagrams near the rods along the trajectory (**b**).

During the loading of the sample, the maximum stresses were localized in the deflection zone of the plate, leading to the initiation of cracks on the outer surface of the plate. The areas of matrix failure are highlighted in red (see Figure 3a). It can be observed that, in the unreinforced plate, the failure field was uniform; however, with the introduction of rods, the area of the polymer matrix exceeding critical stresses decreases.

The stress distribution curves in the reinforcement zone were plotted along a path that passes through the center of the rods in the central cross-section of the sample (as shown in Figure 3b). For comparison, a curve representing the trajectory of the rod placement for the plate without reinforcing elements is also included.

**Figure 4:**
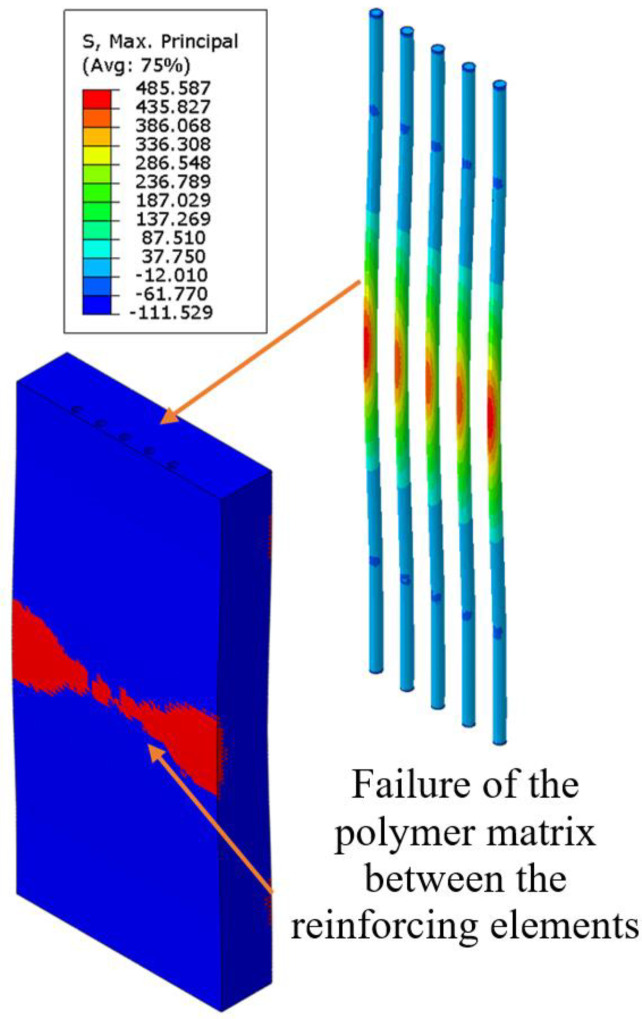
Fields of maximum principal stress in the carbon rods and failure criterion in the polymer matrix of a sample with 1.9 mm between the centers of the carbon rods.

An analysis of the stress distribution showed that maximum stress occurred in the unreinforced polymer material and exceeded the material’s strength limit. The sharp stress gradient on the diagrams corresponded to the arrangement of the rods in the polymer matrix. The reinforcing elements were bearing the primary load, significantly enhancing the strength properties of the plate. However, as the distance between the rods increased, the effectiveness of the reinforcing elements decreased. The strength limit of the rods has not been reached; however, cracks may initiate in the polymer matrix between the reinforcement rods (see Figure 4).

Minimizing the amount of reinforcing material is essential for creating cost-effective sockets, as it also helps prevent excessive weight gain. Therefore, it was necessary to find the optimal distance between the rods, allowing them to be spaced as far apart as possible without compromising the strength of either the rods or the polymer matrix. This ensures a balance between the structural strength and weight characteristics of the socket. To determine the optimal reinforcement spacing, a criterion for assessing strength prior to material failure was introduced as follows:

(3) $$n=\frac{\sigma_{critical}}{\sigma_{max}}\geq 1,$$

where $\sigma_{critical}$ is the material strength limit, and $\sigma_{max}$ is the maximum stress obtained during numerical calculation. Failure in the material occurs when n is less than 1. The results of the material strength assessment are summarized in Table 2. It can be observed that failure of the carbon rods did not occur, while failure of the polymer happened at a distance of 1.9 mm between the centers of the rods. To ensure a small safety margin for the polymer matrix, the value of the criterion n for the polymer matrix was set to 1.1. Therefore, the maximum distance at which the rods can be placed will be 1.5 mm between the centers of the carbon rods.

**Figure 5:**
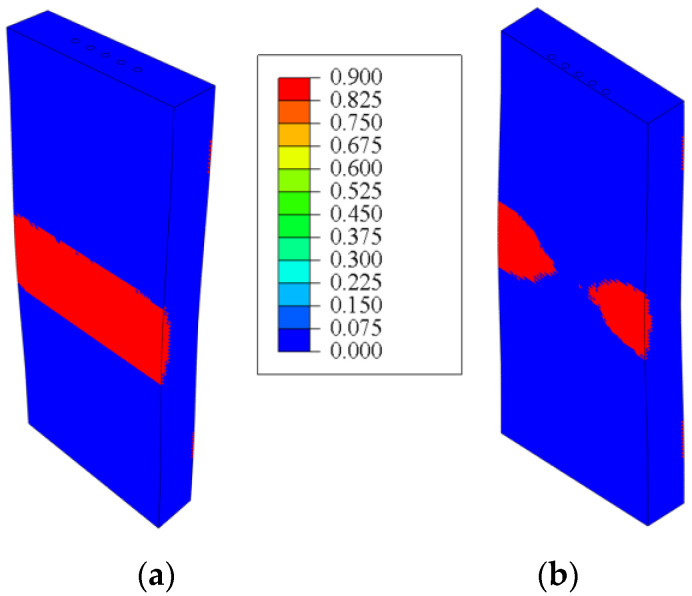
Fields of the failure criterion: (**a**) rods are positioned at the center of the plate, (**b**) rods are positioned at a distance of 0.5 mm from the outer surface of the plate to the center of the rods.

It is worth noting that the rods positioned at the center of a sample did not provide sufficient effectiveness for reinforcement, as failure began on the outer surface (see Figure 5). Thus, the reinforcement rods should be positioned closer to the points of load, at a distance of 0.5 mm from the outer surface of the plate to the center of the rods. This distance was determined based on the numerically simulated damage field observed for the plate without reinforcing elements, by assessing its propagation into the depth of a sample.

**Figure 6:**
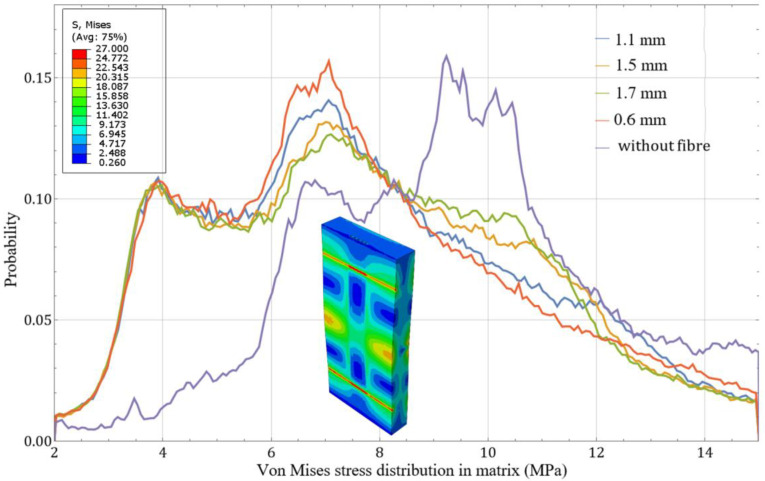
Smoothed histograms for von Mises stress distribution density in the polymer matrix.

To establish reinforcement parameters, the failure field patterns observed on the polymer sample surfaces were evaluated. Simultaneously, a volumetric stress distribution density analysis was employed to assess internal matrix stresses (Figure 6). This quantitative approach utilizes the statistical analysis of random variables, where stress probability within specific ranges is represented as a volumetric histogram. To create such histograms, stress field values were measured in each finite element and weighted by the volumetric contribution of each element. The resulting smoothed histograms visualize stress concentration probabilities and enable the quantitative characterization of stress distribution patterns throughout the entire volume of the computational model.

These results indicated that the presence of reinforcing fibers reduced the overall stress levels in the polymer material. The reinforcement effect intensifies proportionally with reduced spacing between adjacent rods.

The study of three-point bending of polymer samples reinforced with carbon rods led to the following results.

1. The presence of reinforcing elements reduced the stress levels throughout the volume of the polymer matrix and prevented crack initiation on the surface of the sample.
2. At a distance of 1.9 mm between the centers of the rods, the strength criterion had a value less than 1, leading to failure of the polymer matrix between the rods.
3. The optimal distance between centers of the rods was determined to be 1.5 mm, which provides a safety margin according to the strength criterion.
4. The effect of reinforcement was observed in a 4 mm thick plate only when the rods were positioned close to the points of load application.

For more complex structures, such as a cylindrical socket, it is rational to use simplified numerical models. An alternative approach involved calculations where beam finite elements based on Timoshenko beam theory with a circular cross-section were used to model continuous fibers. Additionally, constraints were applied to consider the fibers as an embedded region within the socket model, utilizing the embedded element technique. This technique was used to link elements that are located within so-called “parent elements”. The parent elements controlled the movement of the embedded elements by restricting their degrees of freedom.

**Figure 7:**
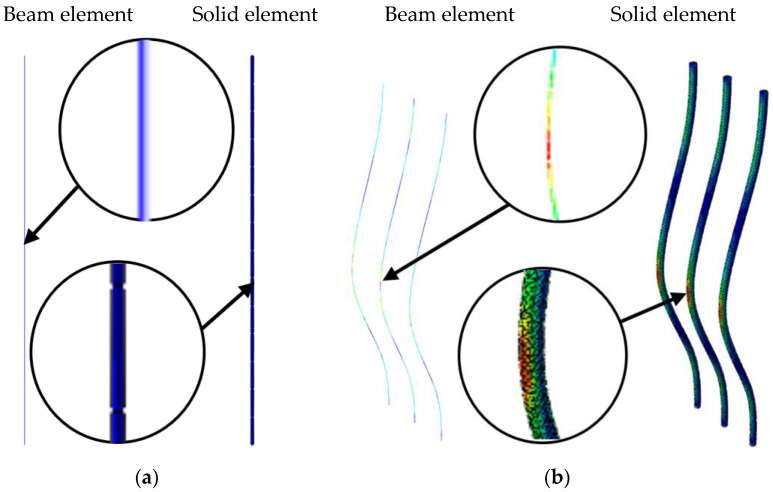
Von Mises stress distribution fields (MPa) in the rods along the load application axis under uniaxial tension (**a**), and in the rods during three-point bending (**b**).

Using beam elements for the carbon fiber rods instead of a set of solid elements required verification through comparison of the two modeling approaches. For this, a uniaxial tension as well as three-point bending problems for the plate were studied separately. In case of uniaxial tension, the load was applied to the end of the rod, defined in terms of displacements, and was equal to 15% of the deformation. The stress fields along the load application axis showed negligible differences in the results for the two calculation methods, both for the uniaxial tension problem and for plate bending (see Figure 7).

In the case of uniaxial tension, the stress fields were homogeneous, both for the case of beam elements, and when using solid elements. The discrepancy in the values of maximum stresses was 0.07%. For three-point bending, maximum stresses were observed at the points of load application, with a discrepancy in the values of maximum stresses of about 1.8%. Thus, the proposed approach employing beam elements provided sufficient calculation accuracy while significantly saving computational time.

### 3.2. Numerical Investigation of Damage Accumulation Process

**Figure 8:**
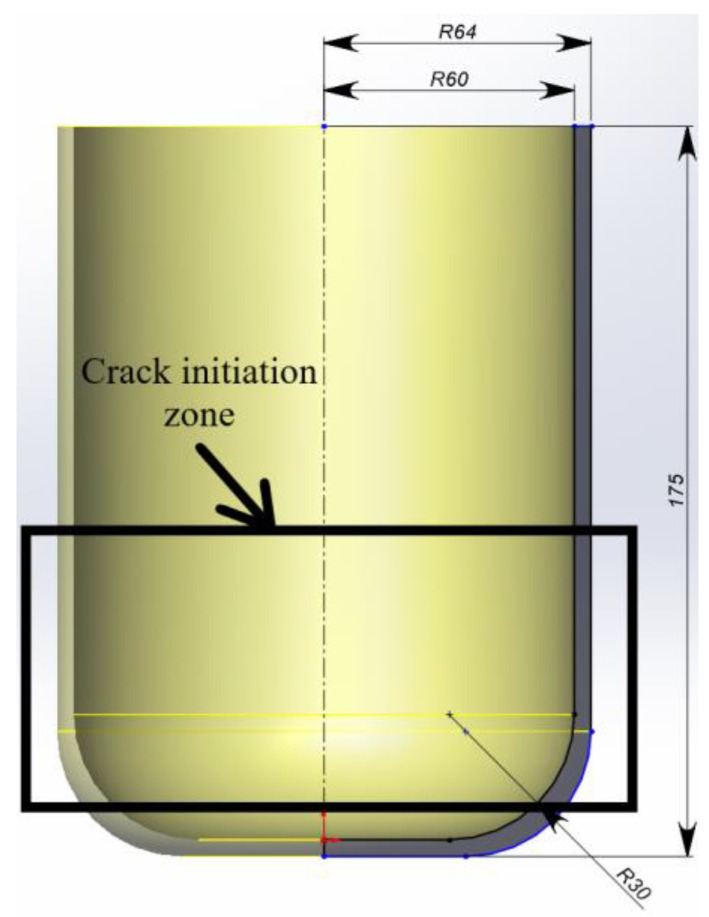
Schematic image of a socket prototype.

Optimizing rod spacing and calculating the distance between the load application point and fibers, while utilizing the beam element method, enables progression to the prosthetic socket’s design phase (see Figure 8).

The analysis assumed non-uniform load distribution from the residual limb across the prosthetic socket’s inner surface. Local reinforcement was strategically applied to the region experiencing peak stress concentrations.

**Figure 9:**
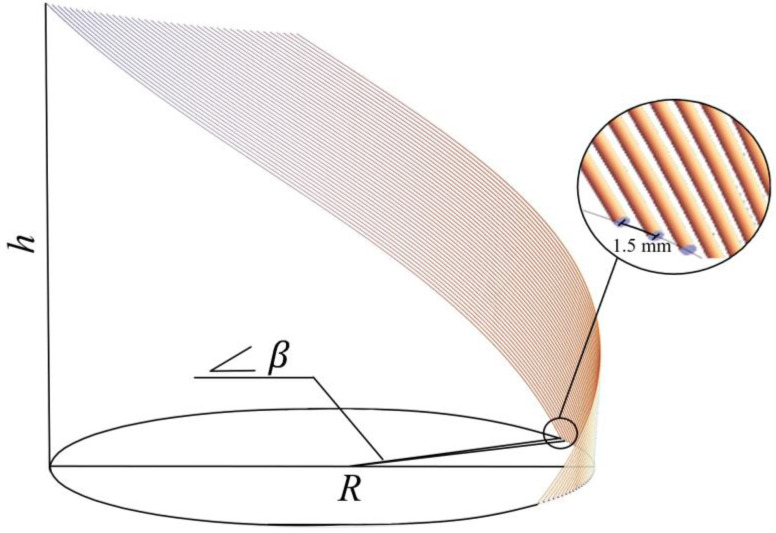
Scheme of the rod geometry (dimensions in mm).

A Wolfram Mathematica code was developed to define the rod configuration in the cylindrical socket, enabling the derivation of reinforcing element trajectories using the following analytical helical line equation:

(4) $$x\left(\varphi \right)=R\cos\left(\varphi \right),\;z\left(\varphi \right)=R\sin\left(\varphi \right),\;y\left(\varphi \right)=\varphi \;R\cot\left(\alpha \right),$$

where *R* represents the radius of the circular arrangement in which the rods are positioned. According to the obtained results, the reinforcement should be located near the load application point, closer to the inner surface of the socket (61 mm). α is the angle of inclination of the carbon rod. The value of this parameter, α=30°, was determined in our previous studies [33,34]. φ is the twisting parameter of the helical line, calculated based on the constraint on the y-coordinate. It is determined depending on the height of the prosthetic socket. The constraints imposed on the *y*-coordinate were based on the height (h = 80) of the reinforcement zone. The resulting array of coordinates was multiplied by the number of rods, and a constraint was set on the angle β between the adjacent rods, calculated from the length of the chord (based on the previously obtained data, the maximum chord length is 1.5 mm). The final output consisted of coordinate sets for each individual rod (see Figure 9). These coordinates were subsequently imported into Abaqus to establish the reinforcement trajectory within the socket model.

**Figure 10:**
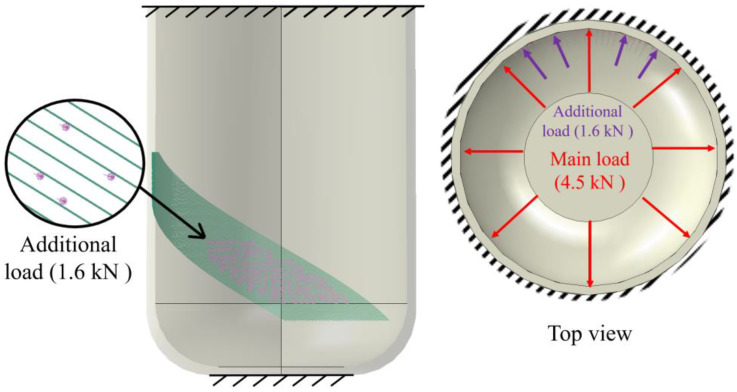
Diagram of boundary conditions and loading conditions.

It was assumed that the socket is tightly fixed onto the limb. Boundary conditions were defined by constraining displacements in all directions along both the upper and lower surfaces of the socket, simulating the prosthetic fixation to the residual limb (see Figure 10).

A maximum load of 4.5 kN (equivalent to the body weight of a 100 kg patient) was uniformly distributed across the entire prosthetic socket surface. This load represented the upper limit of stress that the prosthesis would experience under full weight of a patient. An internal pressure artificially created a zone of high stresses, simulating the uneven load transfer from a stump. To achieve this, an additional load was applied to a section of the socket, with the load value determined from the condition of crack initiation in the un-reinforced model. By iteratively increasing the load value, it was determined that a load of 1.6 kN led to failure of the socket.

In [3], it was proven that additively manufactured polymer sockets exhibit a brittle type of failure, with crack propagation occurring parallel to the printing direction. Therefore, in the numerical implementation of the damage accumulation process, a progressive failure method was used, applying the criterion based on maximum normal stresses. The material’s strength limit was set as a failure criterion; once this limit was reached, the elastic properties degrade. The damage variables in the direction of load application were equal to 0.9, which corresponded to an instantaneous reduction of the mechanical properties of the polymer material by 90% from its original value, according to the brittle type of fracture [35].

**Figure 11:**
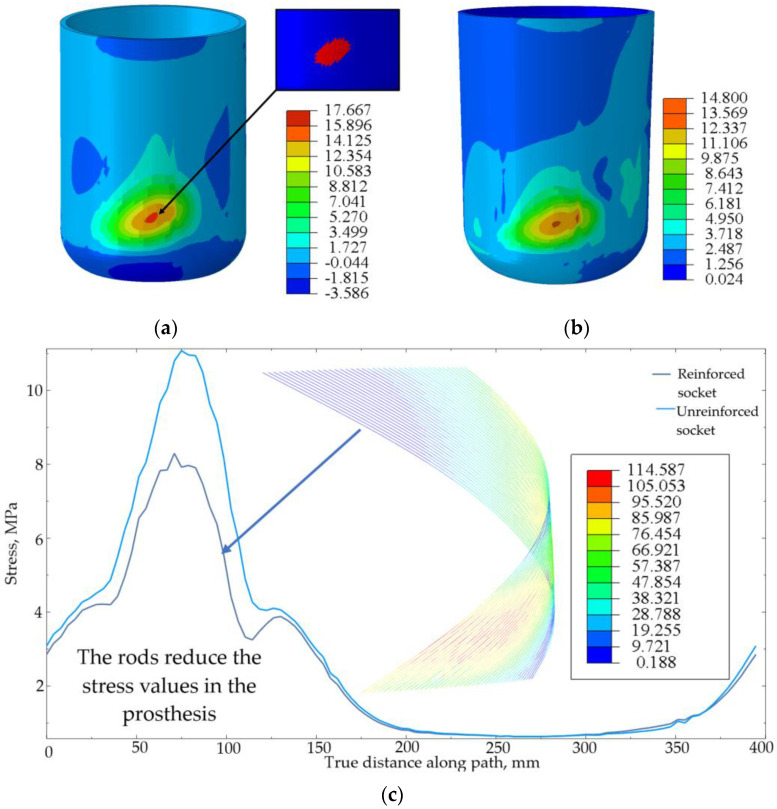
(**a**) Distribution of stress fields and the failure criterion field at the crack initiation stage in the unreinforced socket. (**b**) Distribution of stress fields in the reinforced socket. (**c**) Stress distribution diagrams.

The analysis of the results of computations revealed that failure criteria were exclusively observed in the unreinforced prosthetic socket model under identical loading conditions (see Figure 11a). The unreinforced socket exhibited a maximum normal stress of 17.67 MPa in the direction of load application, surpassing the material’s strength limit. By contrast, the reinforced socket demonstrated a reduced maximum normal stress of 14.80 MPa (see Figure 11b). Thus, the presence of reinforcing rods reduced the stress in the prosthesis by 16.2%. This relationship is clearly illustrated in Figure 11c. The graph illustrates stress distribution curves along a circular path with a 61 mm diameter, which traverses the cross-section of the socket and intersects the centers of the reinforcing rods.

The maximum stress in the rods (114.59 MPa) remained well below their strength limit. Stress distribution analysis revealed peak values concentrated in the load application zone (see Figure 11c). Reinforcement elements in the potential crack initiation regions enhanced the structural stiffness. A numerical failure analysis demonstrated that the unreinforced polymer matrix would fail at its material strength limit, while the reinforced configuration prevented crack propagation, thereby extending the socket’s lifespan.

### 3.3. Manufacturing of Socket Prototype

**Figure 12:**
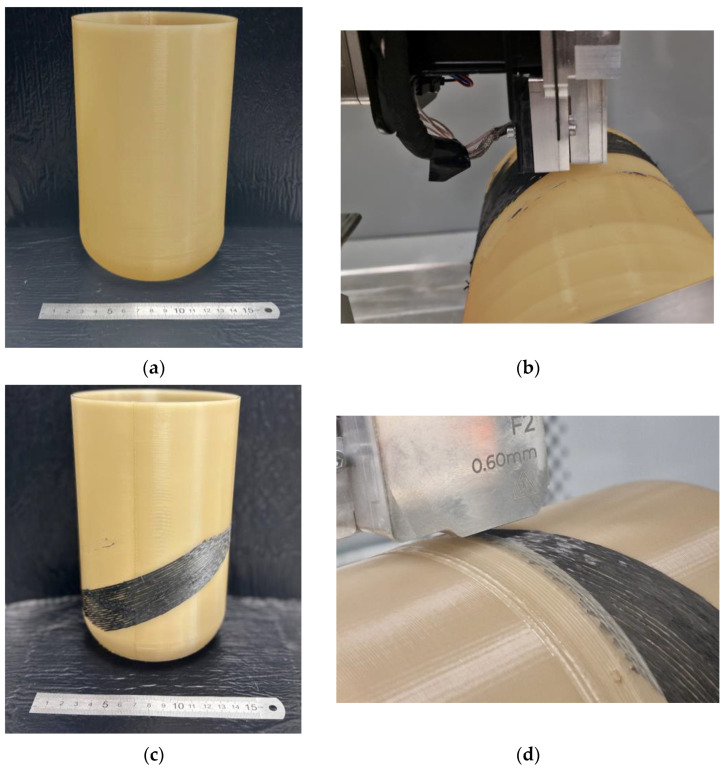
The stages of socket prototype manufacturing: (**a**) thin-walled 3D-printed PA12 cylinder; (**b**) deposition of the carbon rods according to the predetermined trajectories; (**c**) a socket prototype with applied carbon rods; (**d**) sealing of the rods and restoration of the wall thickness with additional layers of PA12.

The socket prototype with the implemented reinforcement was created in three steps. First, a prototype made of PA12 with half of the intended wall thickness was printed using a conventional FFF technique. The controlling G-code algorithm was created using the ideaMaker v5 software (Raise3D, Lake Forest, CA, USA), via a standard slicing sequence (Figure 12a). The filament-placing printing head of the robotic cell worked in the standard planar regime to create this part.

The next stage involved non-planar reinforcement of a prototype with individual carbon rods following the trajectories developed during numerical simulation. The head for deposition of the polymer-coated carbon fiber was used with the additional rotary-tilting positioner, controlled by a specialized controller that provided simultaneous control of all axes, as well as the movement of the actuator, extrusion of the polymer filament, extrusion, and cutting of the carbon rods (see Figure 12b,c). The control program was developed to implement the desired reinforcement geometry governing the above-mentioned parameters. It should be noted that the developed scheme of reinforcement is possible to apply only in the non-planar printing regime.

The last stage involved sealing the carbon rods with additional layers of PA12 and finalizing the part by restoration of the required thickness of the socket’s wall. This was performed in the multi-axis FFF printing mode as new thermoplastic materials had to be extruded on top of the reinforced fibers (see Figure 12d). To implement this, the FFF printing head was oriented orthogonally to the wall of a cylinder using the tilt-rotary manipulator. The custom controlling algorithm was written to perform such non-planar manufacturing mode.

The time used for printing of the cylindrical socket using traditional planar 3D-printing process was about 12 h. The localized non-planar reinforcement following the predetermined rod trajectories takes from 30 min to 1 h depending on the size of the strengthening zone. The following finishing by sealing of the reinforcement may take another 12 h, depending on the designed wall thickness. Thus, with the consequent optimization of the process parameters, the overall manufacturing time may take from 24 to 30 h.

## 4. Discussion

Multi-axis printing is a promising technology for producing prosthetic sockets with reinforcement, allowing for the creation of complex spatial structures. However, despite its advantages, such process can be associated with several technological complexities. The approach with continuous carbon fibers has already been presented in [13,36], but the authors noted the weak adhesion between the polymer material and the individual fibers. The main reason for this issue was the difference in viscosity between the molten matrix and the fibers, leading to poor diffusion bonding. The use of carbon prepreg rods in 3D-printed prostheses can eliminate this problem, as the rods were already impregnated with polymer material, and multiple continuous fibers evenly distribute the load throughout the rod.

**Figure 13:**
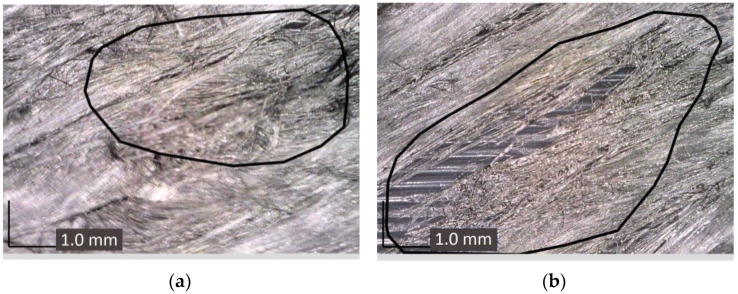

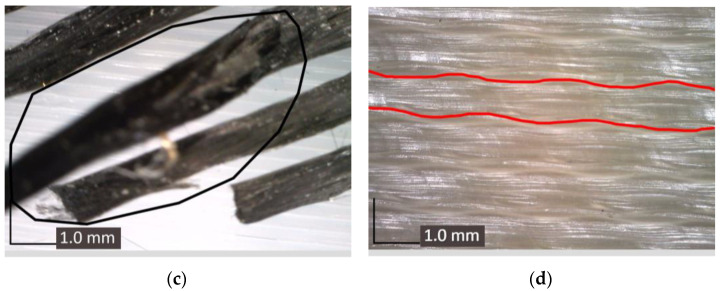
Defects occurring during different stages of multi-axis printing with reinforcement: (**a**) failure of the polymer matrix and carbon fibers of the rod; (**b**) fiber breakage; (**c**) delamination of the fiber; (**d**) over-extrusion of PA12.

One of the most common problems in the reinforcement of 3D-printed parts is fiber breakage. This can occur due to incorrect printing parameters or fiber quality, leading to a decrease in the strength and stiffness of the finished product. The creation of the socket involved multiple stages (as detailed in Section 3.3). During the non-planar reinforcement of the prototype with individual carbon rods along the trajectories, the polymer rod experienced significant bending. This resulted in the rod being cut by the extruder nozzle (see Figure 13a,b). The second most common problem is fiber delamination, which can occur due to insufficient adhesion between the fiber and the polymer matrix (see Figure 13c).

During multi-axis printing, over-extrusion can become an issue, resulting in excess material that forms unwanted bulges or defects on the surface. This problem typically emerges in the final stages, particularly when the carbon rods are covered with additional layers of PA12 (as shown in Figure 13d). These defects not only compromise the appearance of the socket but impose additional stress on the material, potentially reducing the durability of the prosthesis.

Minimizing defects and improving the quality of the prosthetic sockets produced using multi-axis printing is possible through the careful selection of optimal printing parameters. It was found that one of the key parameters for eliminating defects is the gap between the printing object and the extruder nozzle. For the first two defects—fiber breakage and delamination from the polymer matrix—it is crucial to determine the optimal range of this gap, as these defects are interrelated. To determine the size of the gap between the surfaces, a feeler gauge with a thickness of 0.1 mm was used. To prevent carbon rod breakage, manufacturing parameters, such as extrusion speed and the speed of the extruder head moving along the print trajectory, play an important role. The problem of fiber delamination from the polymer matrix can be resolved by increasing the nozzle temperature. A heated rod promotes better adhesion with the polymer material, improving the quality of the reinforcement and reducing the risk of defects.

**Figure 14:**
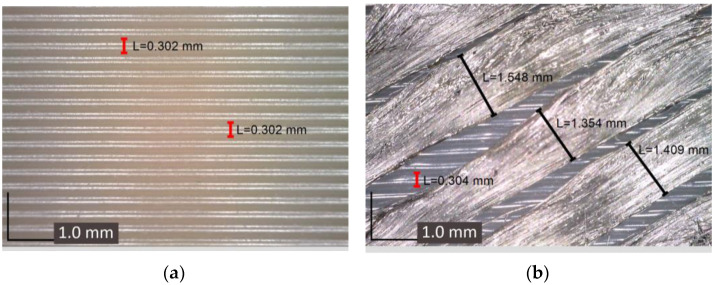

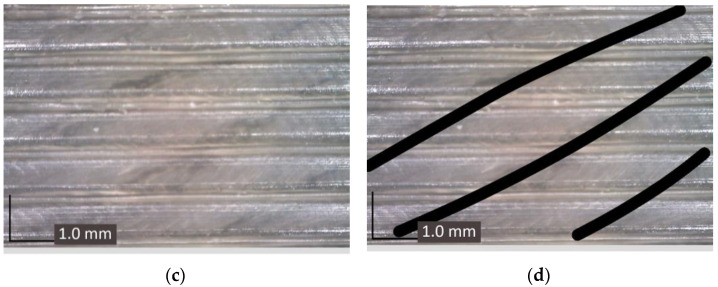
Microstructure of the prosthetic socket at various stages of the printing process: (**a**) initial prototype of the socket; (**b**) laying of carbon rods; (**c**) the rods covered with a layer of polymer material; (**d**) highlighted positioning of the rods.

During this study, these aspects were carefully considered, leading to the creation of a prosthetic socket with a minimal number of defects (see Figure 14a–d). The extrusion speed of the carbon rods varied from 100 mm/min to 500 mm/min, and an optimal speed of 300 mm/min was chosen. The extruder temperature ranged from 265 °C to 300 °C. Temperatures below 265 °C resulted in low adhesion between the prototype and the carbon rod, negatively affecting the reinforcement strength. On the other hand, temperatures above 300 °C caused overheating of the thermoplastic binder, which could lead to its degradation and even burning. The optimal gap for reinforcing the socket with carbon rods of 0.4 mm in diameter was established to be 0.3 mm. This gap ensured effective reinforcement and minimized the risk of defects, such as fiber breakage or delamination from the matrix. This study’s results confirm the effectiveness of selecting optimal printing parameters to enhance the quality of prosthetic sockets.

The presence of gaps between the individual rods can be observed in the microstructure. The irregularity of these gaps is due to the multi-stage process of manufacturing the socket. Initially, the socket is produced in the planar mode, and then the rod is applied in the multi-axial regime. Surface irregularity can cause uneven rod placement. Additionally, minor deformations may occur during the extrusion of the carbon rod, altering its configuration (an issue that requires further investigation and will be addressed in the future). To enhance the quality of rod application, it is suggested to add an extra layer before placement and generate a surface map prior to printing. This approach will help mitigate the effects of surface inhomogeneities on the fiber placement process.

The results of this study indicate that continuous carbon fiber rods provide a more uniform load distribution and significantly increase the strength of the structure. This allows sockets to better withstand mechanical loads, which is a key criterion for their durability. The progressive failure method based on the elastic properties degradation allows for prediction of the damage accumulation and failure processes. The crack initiation process originates in the distal region of the socket and progresses along the lateral surface of the prosthetic device. A numerical analysis of the crack initiation criteria shows qualitative agreement with the experimental results from the prior reported studies [3,4].

It is worth noting that, in addition to carbon fiber reinforcement, another method exists for strengthening the targeted areas of the prosthesis: a composite patch made of the woven material. This approach is often used for 3D-printed structures [37,38,39], including weaves made from carbon fibers. While woven composites enable high-strength performance in the targeted socket regions, their implementation introduces several trade-offs, such as increased material consumption and directional-dependent mechanical properties, which complicate design workflows and may lead to structural unpredictability. These drawbacks may constrain the adoption of woven composites in prosthetic socket design, despite their theoretical benefits. Unlike conventional methods, controlled reinforcement using thin carbon rods enables targeted strengthening of the high-loaded or weak zones.

The mass of the polymer socket can be determined by using the volume of the unreinforced prosthesis, the volume occupied by the rods, and the material density values presented in Table 1. The unreinforced prosthesis had a mass of 0.321 kg, while the reinforced version had a mass of 0.322 kg. This negligible difference indicates that reinforcement in high-load areas provides structural support without significantly impacting the lightweight design of the prosthesis.

The results obtained provide a foundation for subsequent research in this field. First, the proposed design methodology and the selection of optimal parameters can be adapted for personalized models of prosthetic sockets that take into account the geometric features of the residual limb. The second direction focuses on a detailed analysis of prosthesis failure mechanisms, emphasizing the contact interactions between the socket and the rods. Finally, experimental studies will be conducted to establish the mechanical parameters critical for modeling the initiation and progression of the failure processes. The mechanical tests will be performed on several prototypes to validate the models, as well as to assess the influence of the manufacturing parameters on the resulting mechanical response under different loading conditions.

## 5. Conclusions

An approach has been established for designing 3D-printed orthopedic transtibial exoprosthetic sockets that incorporate localized integrated carbon fiber reinforcement. The progressive failure model analysis of the socket’s brittle fracture demonstrated that the reinforcing elements mitigate stress concentrations in the prosthesis, thereby enhancing structural integrity and preventing socket failure. The carbon fiber rods were modeled as structural components with distinct material properties compared to the polymer matrix, employing a beam element-based mesh modeling approach. This study also established optimal reinforcement parameters and determined the preferable rod spacing.

Algorithms and software tools were developed to produce the designed reinforced structure using a multi-axis dual extrusion non-planar additive manufacturing technique. The extrusion speed of the carbon rods, extruder temperature, and gap size between the components were optimized to improve printing quality.

Future research in this area could establish a novel framework for personalized prosthetics through additive manufacturing and multi-axis non-planar reinforcement, enabling rapid customization of the reinforcement parameters to match the individual patient’s needs while predicting the mechanical performance.

## Figures and Tables

**Table 1:** Mechanical properties of materials.

Material	Elastic Modulus E, MPa	Poisson’s Ratio ν	Tensile Strength σ, MPa	Density (g/cm^3^)	Source
Carbon rod	60 000	0.35	520	1.24	From manufacturer
PA12	1445.15 ± 111.18	0.3	15.47 ± 1.40	1.02	[31]

**Table 2:** Determination of the optimal distance between the rods.

Distance Between Centers of Rods, mm	Maximum Normal Stresses in Rods, MPa	Maximum Normal Stresses in Polymer on the Outer Surface of Samples, MPa	n, Rods	n, Polymer
0.6	385.04	10.92	1.35	1.42
0.7	401.91	11.70	1.29	1.32
0.8	415.64	12.15	1.25	1.27
0.9	423.23	12.40	1.23	1.25
1	439.89	13.46	1.18	1.15
1.1	448.30	13.52	1.16	1.14
1.2	446.90	13.62	1.16	1.14
1.3	451.20	13.26	1.15	1.17
1.4	460.18	13.72	1.13	1.13
1.5	461.71	13.89	1.13	1.11
1.6	467.45	14.73	1.11	1.05
1.7	474.58	15.08	1.09	1.03
1.8	477.56	15.17	1.09	1.02
1.9	485.59	15.53	1.07	0.99
2	512.61	16.70	1.01	0.92

## Data Availability

The data presented in this study are available on request from the corresponding author due to privacy restrictions.
